# Depressive disorder comorbid with problematic alcohol use

**DOI:** 10.1192/j.eurpsy.2023.1374

**Published:** 2023-07-19

**Authors:** H. Berrada, H. Chebli, F. Azraf, F. El Omari

**Affiliations:** hôpital psychiatrique arrazi de salé, sale, Morocco

## Abstract

**Introduction:**

Alcohol use disorder is a real public health problem in the world, it consists of a pathological mode of consumption which is characterized by a loss of control and craving. Depressive disorder and alcohol use disorder are among the most frequent mental pathologies and are often associated.

The links between these two disorders can be of several types: Alcohol Induced Depressive Disorders, Primary Depressive Disorders and Secondary Alcohol Dependence. They can also have two-way relationships or be favored by common factors.

**Objectives:**

The objective of our work is to analyze the causal links between alcohol use disorder and depression.

**Methods:**

provide an update via two clinical vignettes and a review of the literature the relationship between alcohol use disorder and depression

**Results:**

The causal relationships between alcohol dependence and psychiatric disorders can be of several types which are not mutually exclusive: primary alcohol dependence, secondary psychiatric disorders, induced by alcohol. This is the case for the majority of depressive disorders encountered in alcohol-dependent patients; primary psychiatric disorders and secondary alcohol dependence; alcohol dependence and anxiety and/or depressive disorders are favored by common factors, in particular personality disorders, encountered in approximately 40% of alcohol-dependent patients. Whatever the direction of causation, alcohol dependence and psychiatric disorders worsen each other over time.Depression and alcohol use disorder are among the most frequent mental pathologies and are often associated.The optimal management of patients with dual diagnosis is so-called “integrated” management, simultaneously combining alcohol and psychiatric therapeutic approaches.

**Conclusions:**

Alcohol consumption impairs the prognosis of depression, increases the risk of suicide, impairs social functioning and increases recourse to the healthcare system.The optimal management of patients with dual diagnosis is so-called “integrated” management. Psychotherapeutic (individual and systemic), drug and psychosocial approaches would be necessary to maximize therapeutic success.

**Disclosure of Interest:**

None Declared

